# Child sexual abuse: report of 311 cases with review of literature

**DOI:** 10.11604/pamj.2015.20.47.4569

**Published:** 2015-01-19

**Authors:** Laila Essabar, Abdenbi Khalqallah, Badr Sououd Benjelloun Dakhama

**Affiliations:** 1 Department of Paediatric Medical Emergencies of Rabat children's Hospital, Morocco; 2 Laboratory of Clinical and Pathological Psychology, Mohammed V University, Rabat, Morocco

**Keywords:** Child, rape, incest, reporting, suicide, depression

## Abstract

Child sexual abuse (CSA) is a global problem that has significant consequences for public health; it has been a prominent topic of public concern for more than a decade, but many basic facts about the problem remain unclear or in dispute. We conducted a study of 311 cases of CSA in order to highlight the epidemiological features and negative impact on victims’ well-being and to emphasize the need for a multidisciplinary approach to the primary prevention and management of CSA. We noted an increase in cases number with male predominance. Most of our patients came from lower socioeconomic classes. The perpetrators were male in 100% of cases; acquaintances in 70% of cases and family members in 22 cases. Physical examination were normal in 61% of cases, however, a range of psychological and physical effects were identified with dramatic health consequences: three cases of attempted suicide, five pregnancies and one case of HIV virus infection.

## Introduction

Childhood sexual abuse (CSA) is a complex life experience that has become the subject of great community concern and the focus of many legislative and professional initiatives. This is evidenced by the expanding body of literature on sexual abuse, public declarations by adult survivors and increased media coverage of sexual abuse issues. However, in Morocco, because sexual abuse is usually a hidden offense, there are no statistics on how many cases actually occur each year. Statistics cover only the cases that are disclosed to child protection associations, to children′s hospitals or to law enforcement. The purpose of our study is to highlight the epidemiological features and negative physical and mental health effects on CSA victims; and emphasize the need for a multidisciplinary approach to the primary prevention and management of CSA.

## Methods

We conducted a 20 years (January 1993- March 2014) retrospective study of CSA victims consulting at the department of paediatric medical emergencies of Rabat children's Hospital. The clinical records of 311 patients were reviewed; we identified demographic data, CSA characteristics, clinical and psychological features and therapeutic and follow-up data.

## Results

### Frequency

**Figure 1:**
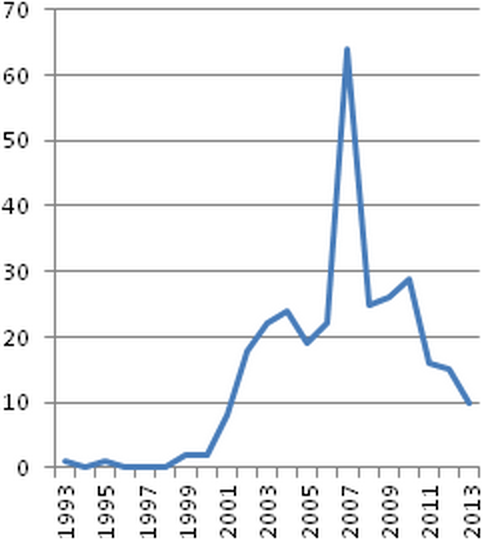
Reported cases by year in our study

Before the late 1999s, CSA cases were sporadic. In the following decades, the number of cases reported annually increased with a peak in 2007, since that year a little decline was recorded (Figure 1).

### Demographic characteristics

**Figure 2:**
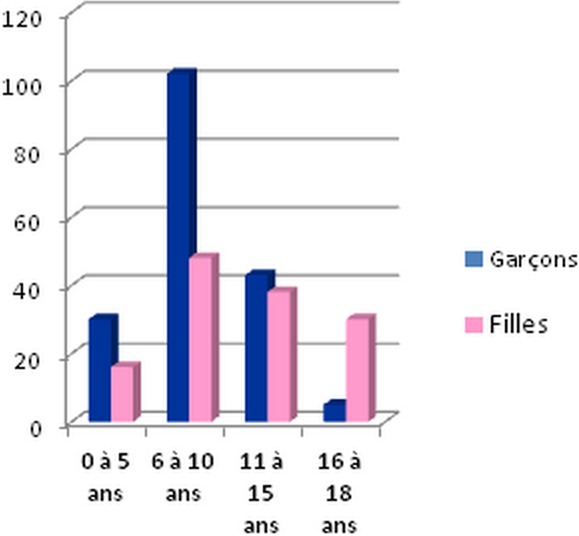
Age and gender of victims in our study

***Age:*** we noted that approximately 15% of victims were between ages 0 and 5 years. Between ages 6 and 10 years, the percentage almost tripled (48%). Ages 11 to 15 years accounted for a quarter (26%) of cases, with children 16 years and older accounting for the remaining 11% of cases (Figure 2).

***Gender:*** before the age of 16 years boys were at about two times higher risk than girls, with a percentage of 68%. Victims 16 years and older were female in 82% of cases (Figure 2).

***Disabilities***: we identified 3 cases of CSA with mental retardation

***Socioeconomic status:*** reported cases came from all socioeconomic classes; however, almost 72% of cases had a low socioeconomic status, the majority was living in sub-rural areas.

***Family structure:*** we observed some cases of familial impairments, in fact parental substance abuse was noted in 11% of cases, the absence of one/both parents was identified in 17% of cases, as well as the presence of a stepfather in the home (8%) and parental conflicts (45%).

### Abuse characteristics

***Offender:*** 100% of child sexual abuse perpetrators were men. Offender's relationship to victim: 81% of victims were sexually abused by a non-relative; offenders outside the family were casual acquaintances of the victim in 70% of cases and strangers in 30% of cases. Employers were the offenders in just 3% of victims. We identified 16% cases of incest; of these, two thirds were abused by their biological fathers. 7% of victims were abused by multiple offenders.

***Type of abuse:*** we noted a spectrum of sexual abuse types ranging from non-contact forms to contact forms of abuse, through to intercourse. In fact, 64% of victims were sodomized, 18% were subjected to fondling and 10% of cases had oral-genital intercourse. We also noted defloration in 8% and exposure to pornography in two cases. Furthermore, the sexual abuse was associated with physical violence in 21% of cases.

***Frequency of abuse:*** CSA occurred as repeated episodes in 67% of cases: of these, victims were reabused by the same perpetrator in 78% of cases.

***Disclosure:*** reporting delay ranges from a few hours to 24 months.

**Physical findings:** clinical examination was normal in 61% of cases; it showed in the remaining cases non specific findings such as vulvovaginitis, erythema, anal fissures and perianal scars, as well as anal dilatation with stool soiling. We noted anal warts in one case. Signs of additional physical violence were noted in 11%.

### Psychological and behavioral symptoms

We observed through our study an array of behavioral disorders with different degrees including fear, anxiety, irritability, regression in school performance, sleep disturbances, eating disorders, social problems as well as poor self-esteem. We also noted inappropriate sexualized in some cases. However, approximately 22% of our patients had no symptom. Incest victims had particularly severe problems such as depression and attempted suicide that was noted in three cases.

### Consequences

The main consequences included three cases of suicide attempts; five cases of pregnancies, three of whom were subjected to incest. Two cases of sexually transmitted infections (STIs) were noted, HIV infection in one case (revealed by the systematic screening for STIs) and HPV anal warts in the second case.

### Management

Management was based on a multidisciplinary approach with on numerous components ranging from medical and psychological treatment to reporting through social support.

## Discussion

### Definition

Having a clear operational definition of child maltreatment - and CSA as a specific aspect of child maltreatment- is increasingly recognized as fundamental to effective preventative strategies [1]. The World Health Organization has defined child sexual abuse as being: “The involvement of a child in sexual activity that he or she does not fully comprehend, is unable to give informed consent to, or for which the child is not developmentally prepared and cannot give consent, or that violates the laws or social taboos of society. Child sexual abuse is evidenced by this activity between a child and an adult or another child who by age or development is in a relationship of responsibility, trust or power, the activity being intended to gratify or satisfy the needs of the other person. This may include but is not limited to: the inducement or coercion of a child to engage in any unlawful sexual activity; the exploitative use of a child in prostitution or other unlawful sexual practices; the exploitative use of children in pornographic performance and materials” [2].

### Statistics

Designing effective child protection measures requires a reliable understanding of the extent of the problem and its context. Globally, the number of studies on the prevalence of CSA has been growing. Based on a summary of existing studies, WHO estimates that between approximately 20 percent of girls and 5 to 10 percent of boys are victims of sexual abuse all over the world [3]. In Morocco, like many developing countries, there is a huge lack in data and the existing findings don't reflect the accurate magnitude of the problem, the main challenge is the sociocultural context and the huge culture of silence that surround sexual issues. A further challenge is that current estimates vary widely as a function of the definitions used, the quality of data collection methods as well as the age of study participants and the age at which childhood is defined. As a global phenomenon, CSA was regarded as rare before the late 1970s. In the following decades, we noted through our study and many other series that the incidence increased dramatically (Figure 1) [4]. Although much of this apparent increase probably reflected a growing awareness among the public and professionals, some studies suggest that the overall incidence of child abuse and neglect increased [5]. The increase in our study may also be due to the creation in 1999 of the children's listening and protection center of the child rights observatory, a structure that provides support and encourages victims to disclose their victimization; reported cases of CSA, however, declined since 2007. This decline could be due to the creation of new medical centers where new cases were referred instead of our department.

### Risk factors

While it is impossible to create a profile of children who will be sexually abused, it is possible to describe characteristics that are more common among victims and are identified as risk factors for CSA.

***Age:*** there is some discrepancy in the available data about whether teenagers are at higher risk or whether the risk is more uniformly distributed. Some data [6] show a relatively uniform risk for children after age 3. Other studies found that over half of the children who were sexually victimized were between 15-17 years old [7]. In our study, nearly half of cases were between 6 and 11years while children aged 16 years and older counted 11% because most of them were referred to the gynecological department (for girls) and the adult emergency department (for boys). Moreover, some studies [8] believe that, as a risk factor, age operates differentially for girls and boys, with high risk starting earlier and lasting longer for girls.

***Disabilities:*** physical disabilities are associated with increased risk [9]. Three factors seem to contribute to this increased vulnerability: dependency, institutional care, and communication difficulties. In a study of 150 interviewed deaf youth at a residential school, 75 children reported being sexually abused, 19 reported being victims of incest, and 3 reported both physical and sexual abuse [8]. We identified, in our study, 3 cases of CSA with mental retardation.

***Gender:*** all reliable studies conclude that girls experience more sexual abuse than do boys in 78% to 89% of cases [10]. Male children in our studies constitute a large proportion of victims before the age of 16 years.

***Socioeconomic status:*** although low socioeconomic status is a powerful risk factor for physical abuse and neglect, it has much less impact on CSA. However, a disproportionate number of CSA cases reported to Child Protective Services come from lower socioeconomic classes [11]. In our study, victims coming from economically disadvantaged backgrounds accounted about three quarters of cases.

***Family structure:*** parental inadequacy, unavailability, conflict, and a poor parent-child relationship show up most consistently in epidemiological studies [12–14] as risk factors for CSA. In many studies children with alcoholic, drug abusing, or emotionally unstable parents are also at risk, as are those with parents who are punitive or distant [15, 16]. However many victims of sexual abuse display none of these markers.

***Other types of victimization:*** children who experience other forms of victimization are more likely to be the target of sexual victimization [7, 17].

**Abuse characteristics *Type of abuse:*** At the extreme end of the spectrum, sexual abuse includes sexual intercourse or its deviations. Yet all offences that involve sexually touching a child, as well as non-touching offenses and sexual exploitation, are just as harmful and devastating to a child's well-being. Touching sexual offenses include fondling; making a child touch an adult's sexual organs; and penetrating a child's vagina or anus no matter how slight with a penis or any object that doesn't have a valid medical purpose. Non-touching sexual offenses include: engaging in indecent exposure or exhibitionism; exposing children to pornographic material; deliberately exposing a child to the act of sexual intercourse; and masturbating in front of a child. Sexual exploitation can include engaging a child or soliciting a child for the purposes of prostitution; and using a child to film, photograph or model pornography. Physical violence is very rarely used; rather the perpetrator tries to manipulate the child's trust and hide the abuse [7, 17]. However in our study CSA was associated with physical violence in 21% of cases.

***Perpetrators:*** the perpetrators of sexual abuse are overwhelmingly male. Male constituted 100% of the offenders in our study and more than 90% in many studies [10, 18, 19]. Although female perpetrators constitute a small percentage; abuse by female has been mushrooming recently [20]. According to studies, the third of convicted sex offenders were sexually abused as children [21]. Our study and several studies agree that approximately half of offenders are acquaintances [6, 22]. The studies differ more about the percentage who are family members, the range is going from 14% to 47% [17, 18, 23] with 16% in our work. Strangers make up the smallest group of perpetrators ranging from 7% to 25% [5, 10, 24, 25] with 24% in our study. The apparent percentage of extrafamilial perpetrators should not obscure the accurate proportion of intrafamilial abuse which tends to be underrepresented among reported cases given the sociocultural restraints surrounding sexual issues especially in developing countries like Morocco.

***Frequency of abuse:*** CSA frequently occurs as repeated episodes that become more invasive with time. Perpetrators usually engage the child in a gradual process of sexualizing the relationship over time [2]. In our study CSA was repeated in 67% of cases: of these, victims were reabused by the same perpetrator in 78% of cases.

**Dynamics of disclosure:** children rarely disclose sexual abuse immediately after the event [26, 27]. Disclosure tends to be a process rather than a single episode and is often initiated following a physical complaint or a change in behavior. Disclosure was delayed in the majority of cases in our study reaching 24 months in a 12 years old incest case.

**Physical findings:** the evaluation of children requires special skills and techniques in history taking, forensic interviewing and examination; the examiner may also need to address specific issues related to consent and reporting of child sexual abuse [28, 29]. In practice, clear physical findings of sexual abuse are seldom seen in children, as physical force is rarely involved. Many studies have found that normal and non-specific findings are common in sexually abused prepubertal girls [30, 31]; clinical examination in our study was normal in 61% of cases. Moreover, in the vast majority of cases the medical examination will neither confirm nor refute an allegation of sexual assault. Clinical examination may reveal physical health consequences [26, 32], that include gastrointestinal disorders (e.g. irritable bowel syndrome, non-ulcer dyspepsia, chronic abdominal pain); gynaecological disorders (e.g. chronic pelvic pain, dysmenorrhea, menstrual irregularities) and somatization (attributed to a preoccupation with bodily processes). Other serious consequences include pregnancy and sexually transmitted infections (STIs), pregnancy was noted in 5 cases in our study, three of whom were incest victims. A study of factors associated with teenage pregnancy [33], found that forced sexual initiation was the third most strongly related factor, after frequency of intercourse and use of modern contraceptives. An organization for teenage mothers in Costa Rica reported that 95% of its clients under the age of 15 had been victims of incest [34]. The prevalence of STIs in pediatric victims of sexual abuse depends on the type of abusive exposure, genital symptoms, prior consensual sexual activity in adolescents, and the regional prevalence of STIs in the adults [35]. Gellert et al [36] evaluated the risk for HIV seroconversion among children with a history of sexual abuse and found that 28 0.4% were HIV seropositive. Systematic screening for STIs in our study revealed HIV in one case. Sexual abuse is the most worrisome form of HPV transmission. One of our patients contracted HPV anal warts.

### Psychological and behavioural symptoms

A variety of adult psychiatric conditions have been clinically associated with CSA. These include the disorders of major depression, borderline personality disorder, somatization disorder, substance abuse disorders, posttraumatic stress disorder (PTSD), dissociative identity disorder, and bulimia nervosa [5]. This apparent diversity can be explained in part by the heterogeneity of CSA experiences, the complexity of the confounds among abuse severity variables, and a host of moderating and mediating constitutional and environmental variables together with important individual differences in coping strategies that may come into play at different points in development in any given case [37]. Some studies suggest that penetration, the duration and frequency of the abuse, force, the relationship of the perpetrator to the child, and maternal support affected the degree of symptomatology [38]. For instance, survivors of incest may have particularly severe problems, especially if the offender was a father or stepfather. 53% of adult survivors of incest said the abuse caused “some”or “great” long-term psychological effects [21]; in our study, incest resulted in three cases of attempted suicide. Numerous studies have found that sexually abused children exhibited more sexualized behaviors than various comparison groups, including non abused psychiatric patients [38–40]. These include such activities as kissing with one's tongue thrust into the other person's mouth, fondling one's own or another person's breasts or genitals, masturbation, and rythmic pelvic thrusting. Furthermore, a history of CSA, but not a history of physical abuse or neglect, is associated with a significantly increased arrest rate for sex crimes and prostitution irrespective of gender [41]. Despite the variety of behavioral disorders that was found in our study, initial psychological evaluation showed no symptoms in approximately 22% of our patients, this result was consistent with those of other studies [38]. The limited longitudinal data available, however, suggest that 10% to 20% of asymptomatic children will deteriorate over the next 12 to 18 months, this phenomenon is termed sleeper effects [5]. Thus, further studies will be needed to find out the long-term effects on our patients.

**Management *medical care:*** includes STIs screening and treatment; decisions about STI testing in children should be made on a case-by-case basis. If testing is warranted, age-appropriate diagnostic tests should be used. Presumptive treatment of children for STIs is not generally recommended [2]. STIs screening in our study was systematic, it was repeated when the abuse occurred recently because STI cultures were likely to be negative.

***Psychological treatment:*** an array of treatment protocols have been offered in the literature providing care for the victims, their families and also the perpetrators. Many studies showed that sexually abused children improved significantly over time [5]. A number of symptoms, especially aggression and sexualized behavior, remain largely resistant to these approaches, however.

***Reporting:*** every community has its own set of laws governing how, and to whom, a report regarding suspicion of child sexual abuse should be made. Typically the reporting law leaves the final determination as to whether or not abuse occurred to the investigators, not the reporters [42]. Morocco like most communities also has a mandatory reporting structure for professionals working with children.

***Counselling and social support:*** provide support to the victim and to those caring him. This may be required even if the child itself is not assessed as needing therapy.

***Follow-up consultation:*** is strongly recommended to ensure that the appropriate counselling referrals have been made and that there is adequate support for the child and family.

## Conclusion

Child sexual abuse has substantial consequences not only for the affected persons, but also for society as a whole, and these can no longer be ignored. This Urgent situation has now been recognized in Morocco which is responding with a diverse range of prevention and intervention programs. However the serious shortcomings in data tend to impede the effectiveness of such measures. Thus, improved studies are required in order to provide data on the accurate magnitude of the CSA, on its distribution and factors that point to vulnerability.
